# Code S: Redesigning Hospital-Wide Peer Review Processes to Identify System Errors

**DOI:** 10.7759/cureus.8466

**Published:** 2020-06-05

**Authors:** Huy D Au, Daniel I Kim, Roger C Garrison, Minho Yu, Gary Thompson, Ramiz Fargo, Brandon Nathaniel, Morteza Chitsazan, Lakshmi K Puvvula, Ali Motabar, Lawrence K Loo

**Affiliations:** 1 Internal Medicine, Riverside University Health System Medical Center, Moreno Valley, USA; 2 Internal Medicine, University of California Riverside School of Medicine, Moreno Valley, USA; 3 Internal Medicine, Loma Linda University School of Medicine, Loma Linda, USA

**Keywords:** peer review, system errors, quality improvement, hospital processes

## Abstract

Hospital medical errors that result in patient harm and death are largely identified as system failures. Most hospitals lack the tools to effectively identify most system errors. Traditional methods used in many hospitals, such as incident reporting (IR), departmental morbidity and mortality conferences, and root cause analysis committees, are often flawed by under reporting. We introduced the Code S designation into our hospital's ongoing physician peer review process as an additional and innovative way to identify system errors that contributed to adverse clinical outcomes. The authors conducted a retrospective review of all peer review cases from January 2008 to December 2011 and determined the quantity and type of system errors that occurred. System errors were categorized based on a modified 5M model which was adapted to reflect system errors encountered in healthcare. The Code S designation discovered 204 system errors that otherwise may not have previously been identified. The addition of the Code S designation to the peer review process can be readily adopted by other healthcare organizations as another tool to help identify, quantify and categorize system errors, and promote hospital-wide process improvements to decrease errors and improve patient safety.

## Introduction

Medical errors commonly occur in the hospital setting and result in patient harm and even death [1-3]. A 2016 study from John Hopkins suggested that medical errors were the third leading cause of death in the US, which translated to an estimated 250,000 or more deaths per year [4]. A large majority of medical errors resulting in patient harm are related to system failures, and not from individual errors or negligence [5-8].

Originally developed by the medical profession to review the qualifications and practice patterns of medical staff physicians [9,10], physician peer review can be used to identify system errors that may contribute to adverse clinical outcomes [11,12]. Unfortunately, the review process to characterize system errors is limited, due to vastly divergent approaches used to conduct physician peer reviews within various healthcare organizations [9,13].

Given the significant negative impact of system-related medical errors on patient safety, many healthcare organizations have established medical error reduction as a priority. This has strengthened the case for developing a systematic process to recognize and subsequently prevent system-related medical errors. The Joint Commission (TJC) maintains that meaningful improvements in patient safety are dependent on each organization’s ability to identify errors and analyze their contributing factors to prevent similar errors from recurring [8].

In 2008, Riverside University Health System - Medical Center (RUHS-MC) modified its hospital-wide physician peer review process to better capture system errors, an approach that RUHS-MC still uses today. All physician peer reviewers were required to report system errors that may have contributed to an unexpected or adverse event [14]. Code S (for "Systems") designation was given to physician peer review cases that identified a contributing system error(s). The purpose of this retrospective chart review study was to (1) demonstrate the feasibility of using the Code S designation in a hospital-wide physician peer review process to identify and track system errors, (2) determine the proportion of reviewed cases that were classified as a Code S designation, and (3) characterize and identify the most common types of system errors identified using our modified 5M model.

## Materials and methods

Hospital description

RUHS-MC is a 439-bed academic safety net hospital and level II trauma center in Moreno Valley, California. The patient population is a low income, predominantly Hispanic population. RUHS-MC is an important site for graduate medical education with residency programs in the specialties of internal medicine, family medicine, anesthesiology, orthopedic surgery, general surgery, neurosurgery, pediatrics, emergency medicine and obstetrics/gynecology. RUHS-MC is also an important site for undergraduate medical education with medical students rotating from three medical schools in the region, University of California, Riverside (UCR), Loma Linda University and Western University of Health Sciences. The medical staff consists of approximately 450 attending physicians.

Peer review process

**Figure 1:**
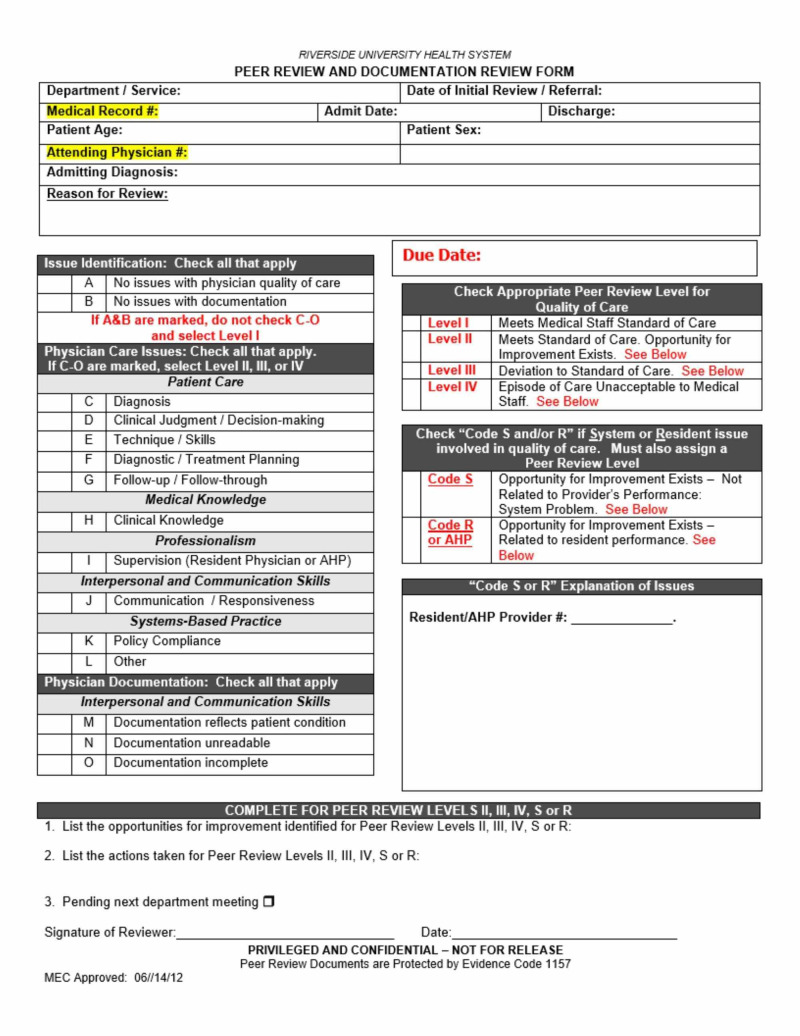
Peer review and documentation review form

The institutional peer review process spans all departments within the hospital and is performed as part of the Ongoing Professional Practices Evaluation (OPPE) system as recommended by TJC [15]. Cases selected for peer review were based on established RUHS policy and included patient deaths, unexpected adverse outcomes, incident reports, quality measure fallouts, routine peer reviews for medical staff re-credentialing, and selected reviews of practitioner performance as determined by the individual departments as recommended by TJC for hospital peer review processes [16]. Physicians providing peer review were medical staff appointed by their respective departments and excluded if they were involved in the case. The peer reviewers completed a standardized peer review form to assign quality of care levels per hospital protocol (Figure 1). In cases where a significant system deficit contributed to a medical error, the peer reviewer would assign a preliminary Code S and document the details of the potential system error(s) that may have contributed to an adverse outcome(s). 

**Figure 2:**
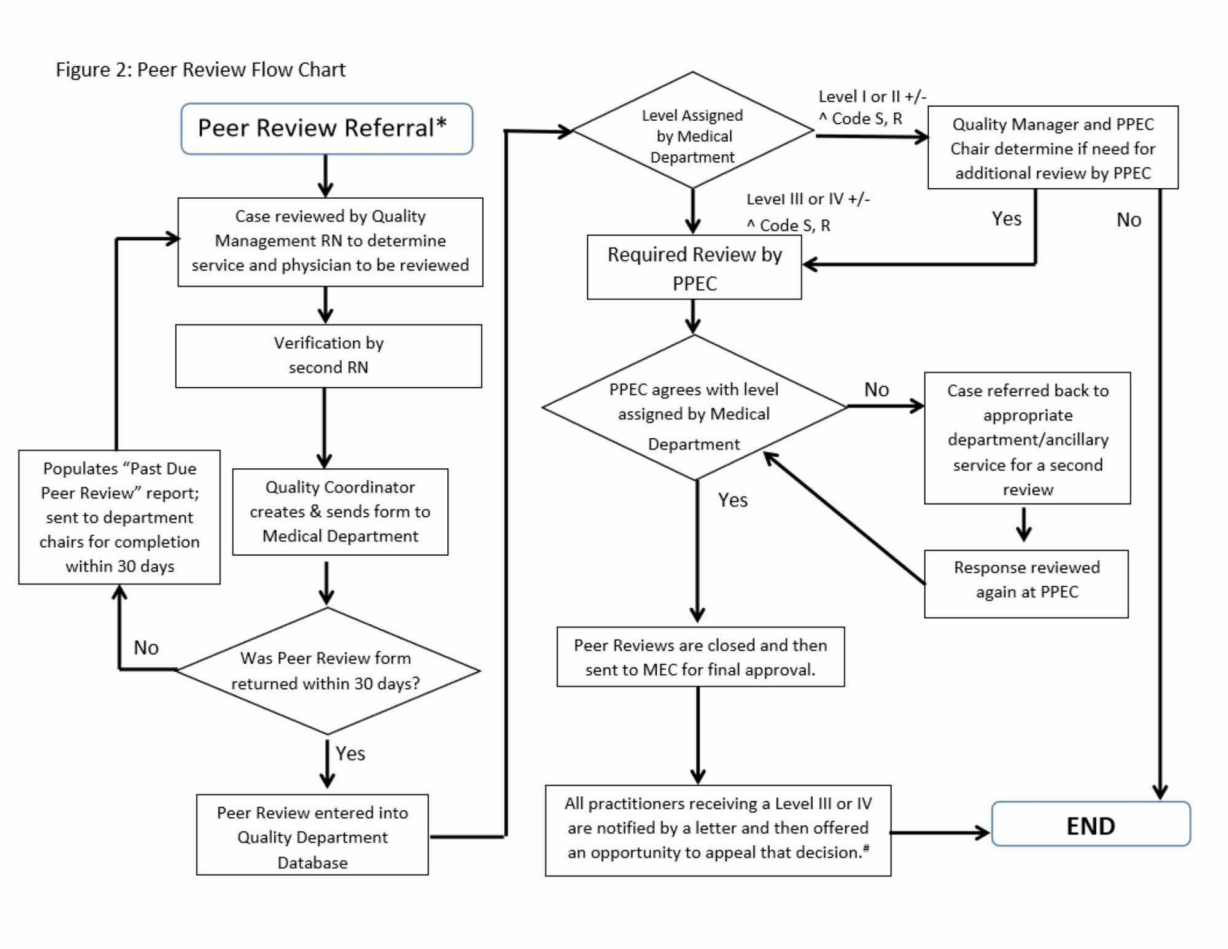
Peer review flow chart PPEC - Professional Practices Evaluation Committee; MEC - Medical Executive Committee

All peer reviews were conducted and adjudicated at the individual department level. After the initial departmental reviews, the peer reviews were forwarded to the Quality Management Department (QMD) and then reviewed by the Professional Practices Evaluation Committee (PPEC), which was comprised of medical staff from multiple hospital departments, QMD, nursing staff and administration. The PPEC was responsible for determining a final quality level including the Code S designation for each peer-reviewed case. If the PPEC disagreed with the initial departmental review, the case was sent back to the department for reassessment. The PPEC also sent inquiries to the appropriate departments to address the system issues identified by the Code S designation. The PPEC closed the review of the Code S once the issues were addressed and corrected (Figure 2). Once the peer reviews were finalized, the information was entered into the QMD database. As the QMD was responsible for performing the root cause analysis, not the initial peer reviewer, very little training was required at the individual provider level.

Study protocol

To evaluate the feasibility and outcome of this peer review process, we conducted a retrospective cross-sectional quality improvement project utilizing peer reviews conducted from January 2008 to December 2011 by the QMD at RUHS-MC. In January 2008, we began assigning Code S as well as Code R (resident) designations to our peer reviews, a process we still utilize today. We selected this date range to coincide with our prior Code R study focusing on potential errors involving resident trainees [14]. The study was reviewed by the RUHS Institutional Review Board and was designated a quality improvement project, which did not require formal review.

Members of the Code S study team evaluated all peer reviews that were assigned a Code S designation between 2008 and 2011 from the QMD database to determine the characteristics of the system errors. Data collected included peer review level assigned, area of deficiency, originating department and whether there was a Code S designation. Two reviewers independently evaluated and assigned the category of deficiency based on a modified 5M framework [7,17]. The reviewers were instructed to review the underlying cause of the deficiency and assign it to one or more of the modified 5M categories. In the cases where the two reviewers assigned different categories to the Code S error, they would discuss the review between themselves and attempt to reach a consensus. In two cases, where consensus was not met between the two reviewers, a third reviewer adjudicated the case to determine the final category assigned.

Our study used the 5M model as a framework to categorize the physician peer reviews with a Code S designation. The 5M model is a well-known risk management tool, primarily used in the manufacturing and aviation industries, to analyze adverse events, and has been successfully adapted to the field of medicine [7,17]. In this study, we modified the traditional 5M model to better reflect the system errors commonly encountered in hospitals.

Man: human factors contributing to the event (e.g. communication, staffing, training)

Machine: tools and equipment used in patient care

Medium: environmental aspects that occur locally within the hospital that contributed to the event

Materials: medications and supplies related to patient care

Management: managerial aspects such as regulations and policies

External Matters: issues outside of the control of the hospital

Data analyses used descriptive statistics including chi-square, Fisher exact, and t-tests. Due to multiple hypotheses testing, the statistical significance was set a priori at p < 0.01.

## Results

We evaluated 8,165 peer-reviewed cases between January 2008 and December 2011. Incomplete peer reviews were removed (four Code S and 353 non-Code S). The final data sets included 204 Code S cases and were compared to 8,106 non-Code S cases.

Table 1 compares the peer-reviewed designations for each of the four levels. For those cases designated Code S, only 23.1% were assigned a Level I (Standard of Care), compared to 88.5% for those designated non-Code S cases. For those cases designated Code S, over 15% were assigned a Level III (Deviation from Standard of Care) or Level IV (Unacceptable Care), compared to less than 1% of those designated non-Code S cases, p<0.0001. The Code S designation was associated with an odds ratio of 23.5 greater likelihood that the two lowest levels of peer-reviewed quality of care (Levels III or IV - “Deviation from Standard of Care” or “Unacceptable Care”) would be identified compared to non-Code S cases. There were no significant differences in the patients’ age or gender among the different departments.

**Table 1:** Comparison of Code S and non-Code S peer review charts and quality of care * Peer Review Levels = I (meets standard of care), 2 (opportunity for improvement), 3 (deviation to standard of care), and 4 (episode of care unacceptable to medical staff). # Numbers may not equal total non-Code S cases due to missing values (but the number of missing values is < 5% of total).

	Code S				Non-Code S				P Value
Number of Cases	204				7812				
Age									
Average + S.D.	38.9 + 22.6				40.2 + 23.5				p = 0.427
Range	0 - 93				0 - 101				
Sex (M/F)									
Number	93 / 111				3469 / 3911^#^				p = 0.69
Percentage	45.6% / 54.4%				52.3% / 54.7%				
Peer Review									
Levels*	I	II	III	IV	I	II	III	IV	
Number	47	123	28	6	6916	830	64	2	p < 0.001
Percentage	23.1	60.3	13.7	2.9	88.5	10.6	0.8	0.02	

**Figure 3:**
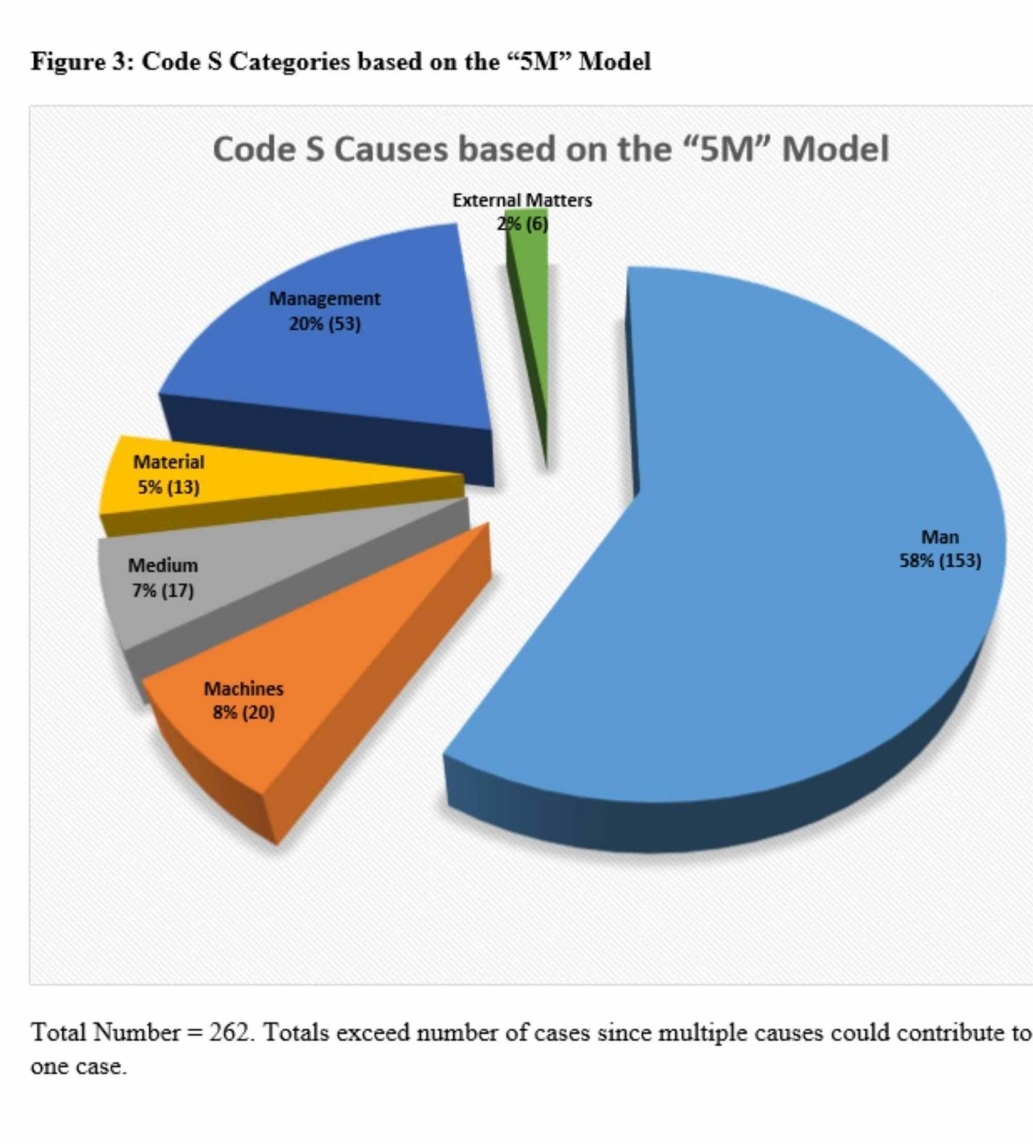
Code S categories based on the “5M” model

Figure 3 categorizes the causes of the Code S error based on our modified version of the 5M model. Among the six causes of system errors, the most common cause identified was “Man” (non-physician human error), which was identified in 58.4% of the cases, while the second most common cause identified was “Management”, accounting for 20.2% of the cases. Table 2 provides a list of examples of the different types of Code S errors.

**Table 2:** Examples of each type of Code S

Table 2: Examples of each type of Code S	
		Man – Communication, staffing, level of training, fatigue	
	Miscommunication between staff members during handoffs
	Surgical subspecialty is only staffed with 1 resident and 1 attending for both clinic and emergent procedures. Emergency surgery had to be delayed until the Attending could be freed up from clinic.
Machines – Tools and equipment used in patient care	
	Ventilator malfunction results in the patient going apneic and subsequently going into a cardiac arrest.
	Consulting service orders a send-out test, which is not reported in the hospital EMR resulting in delays in making the diagnosis and initiating the treatment.
Management	
	Patient coded in the MICU but Anesthesia team did not respond assuming the MICU team would handle the airway. Both MICU Attendings were occupied in procedures and could not respond to the code resulting in a Medicine resident being the most senior person to manage the advance airway insertion.
Materials – Medications and supplies	
	The OR ran out of Urologic irrigate during a procedure resulting in the OR staff trying to make a similar solution during the case. Normal saline was used instead of sterile water resulting in electrolyte abnormalities postoperatively.
Medium – Uncontrollable events	
	Extremely high patient volumes during flu season resulting in Emergency Department delays for the average patient.
	Drunk driver crashes into the power lines for the hospital resulting in the hospital being dependent on the emergency generators. Available computers become disabled resulting in delays with routine care.
External Matters – issues outside the control of the hospital	
	Paramedics did not appropriately start CPR on a patient prior to the patient’s arrival to the hospital.

## Discussion

Approximately 85% of medical errors that occur in hospitals are related to system errors [5-8]. To identify these system errors, hospitals have traditionally relied on limited and confidential forums, such as departmental morbidity and mortality conferences and root cause analysis committees [4].

More recently, hospitals have also implemented the use of incident reporting (IR) systems to help identify patient safety risks related to system errors. Unfortunately, despite these efforts, IR systems capture only a small fraction of total patient safety events, with as few as 7% of all events being reported through IR systems [18].

Chassin et al stated “newer and more effective strategies and tools are needed to identify system errors and to address the complex quality and patient safety challenges facing hospitals today” [19]. Hospital-wide physician peer review processes already exist in hospitals to track provider performances; however, they are not routinely used to identify hospital system errors that contribute significantly to adverse patient outcomes or create potential risks for future events. 

Another innovation is our modification of the 5M model, commonly used in other industries, which allows it to be more relevant for identifying and categorizing medical errors. Our study found the most common classification of system error was “Man”, which represented 58.4% of the total Code S categories. This finding is supported by existing literature indicating that ineffective communication among health care professionals is the leading cause of medical errors and patient harm and emphasizes the importance of creating strategies to enhance teamwork and communication in the hospital setting [20]. 

Our results demonstrate the feasibility of integrating a Code S designation into the hospital-wide physician peer review process to provide an additional tool to identify system errors. This innovation goes beyond the traditional methods available to hospitals to identify and prevent system errors and represents a novel way to identify system errors that may otherwise go unrecognized. We reviewed the first three years of the data since the Code S designation was instituted starting from 2008 to 2011. This program has been very successful and continues to lead to systems changes to date. While this method is not designed to identify all hospital system errors, it is a tool that can be easily integrated into an existing peer-review process to help identify system errors that other hospital processes may not identify. 

Our study also demonstrates a potential gap in existing physician peer review processes. An adverse event could be incorrectly attributed to substandard physician care; when, in fact, a system-related medical error may be involved. For instance, a physician may have ordered a medication for the wrong patient. Upon further investigation, it was discovered that the electronic medical record (EMR) system was down, and the physician ordered the medication on paper without the correct patient identifiers. In this case, the physician peer review would indicate a Level IV with a Code S designation, since the system certainly was a contributing factor to the medical error. In our opinion, the use of the Code S designation when performing peer reviews can serve to unmask system errors that were previously unrecognized.

This interdepartmental multidisciplinary system has the added benefit of allowing for a birds-eye view of errors within the hospital instead of having system errors and possible solutions siloed within individual departments. As the QMD and PPEC oversee information from all individual departments, they can more readily review and identify interdepartmental issues and trends. One such example is when three cases of shoulder dystocia occurred and physical therapy was not consulted by the Pediatrics department. A root cause analysis identified that Labor and Delivery had changed to a different informatics system where the physical therapy evaluation was not incorporated in the documentation. The Labor and Delivery nurses were educated to include discussion of shoulder dystocia as a requirement for Labor and Delivery/post-partum handoff. 

In addition, our finding that Level III (Deviation to Standard of Care) or Level IV (Unacceptable Care) designations were 24 times more likely when a Code S was designated, may suggest that system errors are typically associated with more serious medical errors and worse patient outcomes. In addition, the Code S designation may suggest the presence of underlying system variables that place physicians and patients in higher-risk situations. The magnitude of this association may indicate the importance of identifying system-related issues, which if corrected, could result in a significant reduction in adverse events.

One limitation is that this study was done at a single institution and may not be representative of other organizations and their peer review processes. Other limitations include potential reviewer bias, a low number of system errors relative to the total number of peer-reviewed cases, and missing peer review levels in our database. 

The Code S classifications may have introduced reviewer bias; however, we attempted to limit this potential bias by having two physicians independently assess each case. In the two cases where a consensus was not met, a third reviewer independently reviewed the case and made a final decision. Also, our protocol included a multidisciplinary peer review process for applying the Code S designations which reduced the potential for single reviewer bias or error. 

While the data showed a low percentage of system errors identified from the total number of peer reviews, we believe the percentage would have been higher if routine peer reviews for medical staff re-credentialing, which significantly increased the peer review denominator, were removed and only the incident triggered peer reviews were analyzed. However, the database did not allow for this type of analysis. 

Another limitation to this process is the possibility of false negatives. The initial reviewer could have failed to identify a system error that contributed to the peer review. However, we attempted to mitigate this by virtue of a step-wise process. After the initial reviewer evaluated the case, it was re-evaluated by the department, voted on and then sent to QMD and PPEC for final review and approval. This process affords multiple opportunities for a system issue to be identified not only by the initial reviewer, but by other physicians in the department, as well as the multidisciplinary QMD and PPEC members. 

While there were missing peer review levels in our database, which were excluded from the analysis, (1.9% with Code S designation and 4.3% without Code S designation), they represented a small percentage of the overall data set and were not felt to have a significant impact on the results.

## Conclusions

System errors are the most common types of errors within hospitals. Despite this fact, most system errors are not rigorously identified and systematically categorized in most hospital-wide peer review processes. Unfortunately, there is no tool that can identify all hospital system errors. Having more effective methods for identifying system errors is the first step that allows hospitals to more effectively combat and reduce system errors. Identification of system errors by peer review can be a helpful adjunct in identifying safety and quality of care issues in hospitals and facilitate system changes to improve patient care and decrease risk for patient harm. To our knowledge, this is the first tool that integrates the identification and evaluation of system errors into the hospital-wide peer review process. The addition of the Code S designation to the peer review process can be readily adopted by other healthcare organizations as an effective tool to help identify, quantify and categorize system errors, and promote hospital-wide process improvements to decrease medical errors and improve patient safety. 
